# Amplitude of Low-Frequency Fluctuation With Different Clinical Outcomes in Patients With Generalized Tonic–Clonic Seizures

**DOI:** 10.3389/fpsyt.2022.847366

**Published:** 2022-04-01

**Authors:** Meidan Zu, Lulan Fu, Mingwei Hu, Xiaoyan Cao, Long Wang, Juan Zhang, Ziru Deng, Bensheng Qiu, Yu Wang

**Affiliations:** ^1^Department of Neurology, The First Affiliated Hospital of Anhui Medical University, Hefei, China; ^2^Department of Pediatrics, The Fourth Affiliated Hospital of Anhui Medical University, Hefei, China; ^3^Department of Neurology, The Second People's Hospital of Hefei, Hefei, China; ^4^Center for Biomedical Engineering, University of Science and Technology of China, Hefei, China

**Keywords:** generalized tonic–clonic seizures, seizure-free, non-seizure-free, amplitude of low-frequency fluctuation, the fusiform gyrus

## Abstract

**Background:**

Generalized tonic–clonic seizures (GTCS) are associated with significant disability and sudden unexpected death when they cannot be controlled. We aimed to explore the underlying neural substrate of the different responses to antiseizure drugs between the seizure-free (SF) and non-seizure-free (NSF) patients with GTCS through the amplitude of low-frequency fluctuation (ALFF) method.

**Methods:**

We calculated ALFF among the SF group, NSF group, and healthy controls (HCs) by collecting resting-state functional magnetic resonance imaging (rs-fMRI) data. One-way ANOVA was used to compare the ALFF of the three groups, and *post-hoc* analysis was done at the same time. Pearson's correlation analysis between ALFF in the discrepant brain areas and the clinical characteristics (disease course and age of onset of GTCS) was calculated after then.

**Results:**

A significant group effect was found in the right fusiform gyrus (R.FG), left fusiform gyrus (L.FG), left middle occipital gyrus (L.MOG), right inferior frontal gyrus (R.IFG), right precentral gyrus (R.PreG), right postcentral gyrus (R.PostG), and left calcarine sulcus (L.CS). The SF and NSF groups both showed increased ALFF in all discrepant brain areas compared to HCs except the R.IFG in the NSF group. Significantly higher ALFF in the bilateral FG and lower ALFF in the R.IFG were found in the NSF group compared to the SF group.

**Conclusions:**

Higher ALFF in the bilateral FG were found in the NSF group compared to the SF and HC groups. Our findings indicate that abnormal brain activity in the FG may be one potential neural substrate to interpret the failure of seizure control in patients with GTCS.

## Introduction

The prevalence of idiopathic generalized epilepsy (IGE) is ~1 per 1,000 adults (1) and accounts for 20 to 55% of all epilepsies (2). Generalized tonic–clonic seizures (GTCS) are one subtype of IGE (3) and are also the most dangerous type of seizures, commonly known as the grand mal, and have received a lot of attention because of the increased risk of injury (4) and sudden unexpected death in epilepsy (5, 6). Most generalized seizures can be effectively controlled with appropriate antiseizure drugs; however, around 20–30% of patients still failed to achieve seizure control, presenting a non-seizure-free state (7). Generally speaking, GTCS are not candidates for curative resective surgical procedures due to the generalized spike-wave discharges during seizures and are also associated with significant morbidity and disability when they cannot be controlled (4, 8). However, emerging evidence suggests that “generalized” epilepsy is not truly general, but rather specific areas of the brain are affected mainly, while other areas are relatively unaffected (9, 10). Therefore, it is particularly important to understand the pathophysiology from a variety of aspects and perspectives in order to obtain more effective treatments for patients with GTCS who failed to achieve seizure control.

Previous studies have shown that different changes were observed in gray matter volumes, regional glucose metabolism, and the electroencephalogram (EEG) in the seizure-free group compared with the non-seizure-free group in other types of epilepsy through surgery or antiseizure drug treatment (11–13). For example, a follow-up study published in *JAMA Neurology* showed that a larger proportion of the piriform cortex was resected in the seizure-free group compared with the non-seizure-free group in neurosurgical treatment of temporal lobe epilepsy (11). Of course, research also showed that the non-seizure-free IGE patients on adequate antiepileptic drug treatment did not present more severe white matter tract involvement compared to the seizure-free patients (14). So far, the underlying neural substrate of these two epileptic clinical outcomes in GTCS remains unclear. Conventional magnetic resonance imaging (MRI) examinations often present normal neuroimaging findings in patients with GTCS. In recent decades, with the development of neuroimaging methods, increasing studies suggested that the abnormal structure and functional connectivity in cortical and subcortical brain regions, like the thalamus, cingulate cortex, and inferior frontal gyrus, were found in GTCS through functional magnetic resonance imaging (fMRI) (15–20). However, most studies aimed at differences between patients with GTCS and healthy controls through fMRI, while there were little reports on the differences between the seizure-free and the non-seizure-free patients afflicted with GTCS.

The fMRI with simultaneous EEG recording studies have found that interictal epileptiform discharges in epileptic patients resulted from a population of abnormally hyperactive and hypersynchronous neurons and that the characterization of spontaneous neuronal activities was based on the fact that there are coherent low-frequency fluctuated blood oxygen level-dependent (BOLD) signals in widespread but functionally related brain regions (21–23). The amplitude of low-frequency fluctuation (ALFF) is just frequently used to reflect resting brain activity by measuring the spontaneous low-frequency oscillations of the BOLD signal of every single voxel in the brain without any predefined seed region of interest (24, 25) and has been widely applied in epilepsy (26–28) and other brain diseases (29, 30). For example, increased ALFF in the epileptogenic regions and decreased ALFF in the default mode regions were found in epilepsy (27). And there were also some studies suggesting that ALFF may be a new biomarker for the physiological state of the brain (31). Therefore, we evaluated the underlying neural substrate of the different responses to adequate antiseizure drugs in patients with GTCS *via* ALFF methods.

In the current study, we collected resting-state fMRI (rs-fMRI) data from three groups of adults, including the seizure-free (SF) group, the non-seizure-free (NSF) group, and healthy controls (HCs). We performed an exploratory whole-brain analysis to identify the underlying neural substrate of these two epileptic clinical outcomes in GTCS through ALFF analysis as the outcome measures because it reflects resting whole brain activity and has strong test–retest reliability (24). Based on the above background in GTCS, we hypothesized that patients in the NSF group may have potential difference of ALFF in the thalamus, cingulate cortex, frontal lobes, and other related brain regions compared to the other two groups, which may be interpreted as the failure of seizure control in GTCS. Besides, the correlation between ALFF values and clinical symptoms was further investigated.

## Materials and Methods

### Participants

The patients in the current study were recruited from the epilepsy clinics of the First Affiliated Hospital of Anhui Medical University in Hefei from August 2019 to February 2020. After that, patient recruitment had to be halted because of COVID-19. They had all received the clinical diagnosis of IGE with GTCS according to the International League against Epilepsy (ILAE) Classification 2017 (32) and clinical interview from two qualified epileptologists based on the clinical grounds and EEG findings. The inclusion criteria for patient recruitment were as follows: (1) bilateral and symmetric generalized motor seizure, like muscle stiffness, severe muscle contractions all over the body, and loss of consciousness at the same time; (2) at least one generalized spike-wave discharge during interictal EEG recording; (3) more than 2 years with disease course of GTCS; and (4) no structural abnormality *via* conventional MRI, like trauma, tumor, or intracranial infection. The exclusion criteria were as follows: (1) pregnancy; (2) substance abuse, other neurologic diseases, or chronic diseases; (3) Mini-Mental State Examination (MMSE) score of <24 points; (4) history of partial seizures; (5) predominant focal EEG abnormalities; (6) falling asleep during rs-fMRI scanning; (7) head motion exceeding 2.0 mm or involved rotation exceeding 2.0°; and (8) structural abnormality at conventional MRI. Two patients were excluded owing to excessive motion (>2.0 mm, 2.0°). Finally, 36 outpatients diagnosed with GTCS were included in this study, and all of them were right-handed and ranged from 18 to 55 years in age. Then, 36 patients with GTCS were divided into two groups based on their clinical presentation: 15 patients had adequate seizure control for at least 2 years classified as the SF group and 21 patients presented at least one recurrent GTCS in the previous 3 months classified as the NSF group before rs-fMRI scanning, despite regular use of the adequate antiseizure drugs.

For HCs, we recruited 27 right-handed healthy participants without a history of seizures who were matched with the GTCS groups in age and education years, maintaining a similar proportion of men and women as in the patient groups. Exclusion criteria were the same as for patients except for the diagnosis of GTCS.

The study was approved by the Anhui Medical University Ethics Committee, and all patients and HCs signed informed consent forms.

### Clinical Assessment

Clinical information including demographic data, education level, past medical history, and use of antiseizure drugs was collected from all patients. The Mini-Mental State Examination (MMSE) was used to evaluate the cognitive function of all the participants (33).

### Neuroimaging Data Acquisition

We acquired rs-fMRI images of participants with the same procedure as described by Zu et al. at the University of Science and Technology of China, Hefei, Anhui Province (34). Specifically, during scanning, all participants were instructed to keep their eyes closed without moving the body and not to think of anything in particular. Functional images were conducted with a 3.0 T MRI scanner (Discovery GE750w, GE Healthcare, Buckinghamshire, UK) composed of 217 echo-planar imaging volumes with the following parameters: repetition time (TR) = 2,400 ms; echo time (TE) = 30 ms; flip angle = 90°; matrix size = 64 × 64; field of view = 192 × 192 mm^2^; slice thickness = 3 mm; 46 continuous slices (voxel size = 3 × 3 × 3 mm^3^). T1-weighted anatomic images with 188 slices were also acquired in sagittal orientation (TR = 8.16 ms; TE = 3.18 ms; flip angle = 12°; field of view = 256 × 256 mm^2^; slice thickness = 1 mm; voxel size = 1 × 1 × 1 mm^3^).

### Data Preprocessing

Functional data were preprocessed with the Data Processing Assistant for Resting-State fMRI Advanced Edition (DPARSFA) V4.3 software package (35), which is a widely used rs-fMRI analytic tool and based on the Statistical Parametric Mapping (SPM8). The first 10 volumes were discarded to exclude the influence of unstable longitudinal magnetization. We processed the remaining volumes with the following steps: (1) slice timing correction; (2) motion correction; (3) co-registration to respective structural images; (4) nuisance regression with 24 Friston motion parameters, white matter high signal, and cerebrospinal fluid signal as regressors; (5) spatial normalization to a standard template (Montreal Neurological Institute; MNI) based on the diffeomorphic anatomical registration through the exponentiated lie algebra (DARTEL) algorithm (36), which provides greater registration accuracy than unified segmentation (37); and (6) spatial smoothing employing DARTEL with a 6 mm at full width half-maximum three-dimensional Gaussian kernel (38).

### **ALFF** Analysis

ALFF analysis was performed using the DPARSFA software after preprocessing. The calculation procedure was the same as reported in previous studies (25, 27). First, with the fast Fourier transform, the time series of each voxel was transformed to a frequency domain, and the square root of the power spectrum was calculated. Then, the averaged square root of the power across 0.01–0.08 Hz was determined as the ALFF measurement. For standardization, the ALFF of each voxel was further divided by the global mean of ALFF values. The standardized ALFF of each voxel then has a value of about 1, and this standardization procedure is analogous to that used in PET studies (39).

### Statistical Analysis

Two patients with GTCS whose head motion exceeded 2.0 mm or involved rotation exceeding 2.0° during the rs-fMRI scanning were excluded. Finally, 15 SF patients, 21 NSF patients, and 27 HCs were analyzed. One-way analysis of variance (ANOVA) was performed with the DPABI software (35) as a measure of the resting-state ALFF difference among the three groups. The result after one-way ANOVA was overlain on the Ch2 template. All statistical maps were corrected for multiple comparisons using the Gaussian random field (GRF) method with the threshold set at a voxel level of *p* < 0.001 in combination with a cluster level of *p* < 0.05, two-tailed, and then the clusters were saved. Then the values of ALFF were acquired by extracting signals at the saved clusters above. Finally, the software SPSS 16.0 was used to analyze the ALFF values of different groups. One-way ANOVA was used to compare the ALFF values of the three groups, and Bonferroni correction of *post-hoc* analysis was done at the same time.

### Correlation Analysis

To investigate the underlying linear association of different ALFF values across the SF and NSF groups, Pearson's correlation coefficients between ALFF values in the discrepant brain areas and the clinical characteristics (disease course and age of onset of GTCS) were calculated.

## Results

### Clinical Data

Thirty-six patients with GTCS and 27 HCs completed all procedures. The basic characteristics of demography and clinical outcomes for the participants are shown in Table 1. There were no significant differences as regards age (*F* = 1.561, *p* = 0.218), gender (χ^2^ = 0.206, *p* = 0.902), education (*F* = 1.320, *p* = 0.275), and MMSE scores (*F* = 2.133, *p* = 0.127) among the three groups. Besides, we did not find significant differences about age of onset (*t* = −0.970, *p* = 0.339) and course of disease (*t* = −0.296, *p* = 0.769) between the SF and NSF groups.

**Table 1:** Demographic and clinical characteristics of participants.

	**SF**	**NSF**	**HCs**	**F/t/χ2**	***P***
Sample size (*n*)	15	21	27	-	-
Age (year)	26.53 (7.63)	30.19 (8.45)	31.13 (11.01)	1.561	0.218
Gender (M/F)	6/9	10/11	12/15	0.206	0.902
Education (years)	13.73 (3.79)	11.81 (4.66)	13.41 (3.52)	1.320	0.275
Age of onset (years)	17.27 (7.40)	20.24 (10.06)	-	−0.970	0.339
disease course (month)	111.20 (93.56)	119.43 (71.85)	-	−0.296	0.769
MMSE	29.67 (0.816)	28.90 (1.81)	29.63 (1.11)	2.133	0.127
**Medications (patient number)**					
Lamotrigine	10	6			
Depakine	6	17			
Levetiracetam	0	1			
Phenobarbital	1	1			
Phenytoin	0	2			
Oxcarbazepine	0	3			
Carbamazepine	0	1		

*MMSE, the Mini-mental State Examination; SF, seizure-free; NSF, non-seizure-free; HCs, healthy controls*.

*One-way ANOVA were used for age, education and MMSE among the three groups. Two-sample t-tests were used for age of onset and disease course between the SF and NSF groups. Chi-square test was used for gender among the three groups*.

*Data are presented as mean (standard deviation)*.

### Group Differences in ALFF

**Figure 1:**
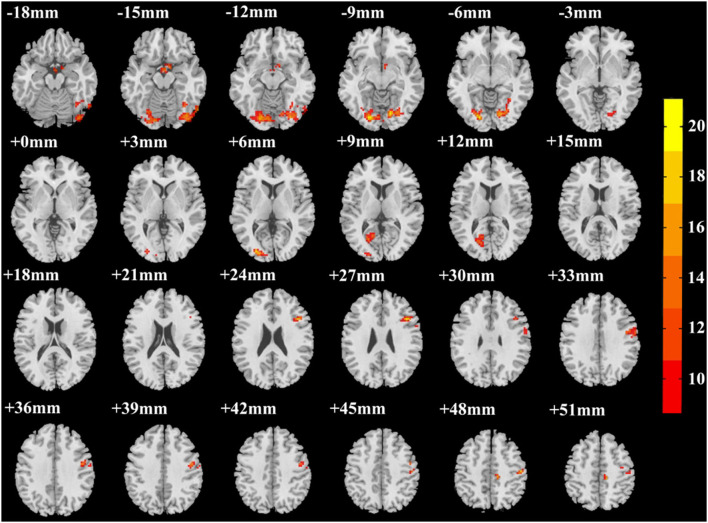
Group differences in ALFF among three groups (SF, NSF, and HCs). A significant group effect was found in the right fusiform gyrus, left fusiform gyrus, left middle occipital gyrus, right inferior frontal gyrus, right precentral gyrus, right postcentral gyrus, and left calcarine sulcus (correction with Gaussian random field, voxel *p* < 0.001, cluster *p* < 0.05, two-tailed).

A significant group effect was found in the right fusiform gyrus (R.FG, *F* = 30.290, *p* < 0.001), left fusiform gyrus (L.FG, *F* = 23.388, *p* < 0.001), left middle occipital gyrus (L.MOG, *F* = 20.437, *p* < 0.001), right inferior frontal gyrus (R.IFG, *F* = 24.357, *p* < 0.001), right precentral gyrus (R.PreG, *F* = 21.650, *p* < 0.001), right postcentral gyrus (R.PostG, *F* = 20.295, *p* < 0.001), and left calcarine sulcus (L.CS, *F* = 15.429, *p* < 0.001) (Table 2; Figure 1).

**Table 2:** One-way ANOVA and pairwise comparison of ALFF among the three groups.

**Brain regions**	**Voxel size**	**Peak coordinates (mm)**			**F value**	***P-*value**	**HCs vs. SF (*p*)**	**HCs vs. NSF (*p*)**	**SF vs. NSF (*p*)**
		**x**	**y**	**z**					
R.FG	188	15	−75	−9	30.290	< 0.001	^**^ < 0.001	^**^ < 0.001	^*^0.028
L.FG	141	−18	−81	−9	23.388	< 0.001	^**^0.005	^**^ < 0.001	^*^0.027
L.MOG	31	−27	−87	6	20.437	< 0.001	^**^ < 0.001	^**^ < 0.001	0.546
R.IFG	31	42	18	27	24.357	< 0.001	^**^ < 0.001	0.095	^**^ < 0.001
R.PreG	43	27	−21	63	21.650	< 0.001	^**^ < 0.001	^**^ < 0.001	1.000
R.PostG	78	51	−18	48	20.295	< 0.001	^**^0.002	^**^ < 0.001	0.155
L.CS	45	−21	−66	9	15.429	< 0.001	^**^0.001	^**^ < 0.001	0.954

*ALFF, the amplitude of low-frequency fluctuation; R.FG, right fusiform gyrus; L.FG, left fusiform gyrus; L.MOG, left middle occipital gyrus; R.IFG, right inferior frontal gyrus; R.PreG, right precentral gyrus; R.PostG, right postcentral gyrus; L.CS, left calcarine sulcus; SF, seizure-free; NSF, non-seizure-free; HCs, healthy controls*.

*All statistical maps were corrected with Gaussian Random Field method with the significance of voxel p < 0.001, cluster p < 0.05, two-tailed*.

** *p < 0.01*,

* *p < 0.05 after Bonferroni correction of post-hoc analysis*.

### Pairwise Comparison of ALFF

**Figure 2:**
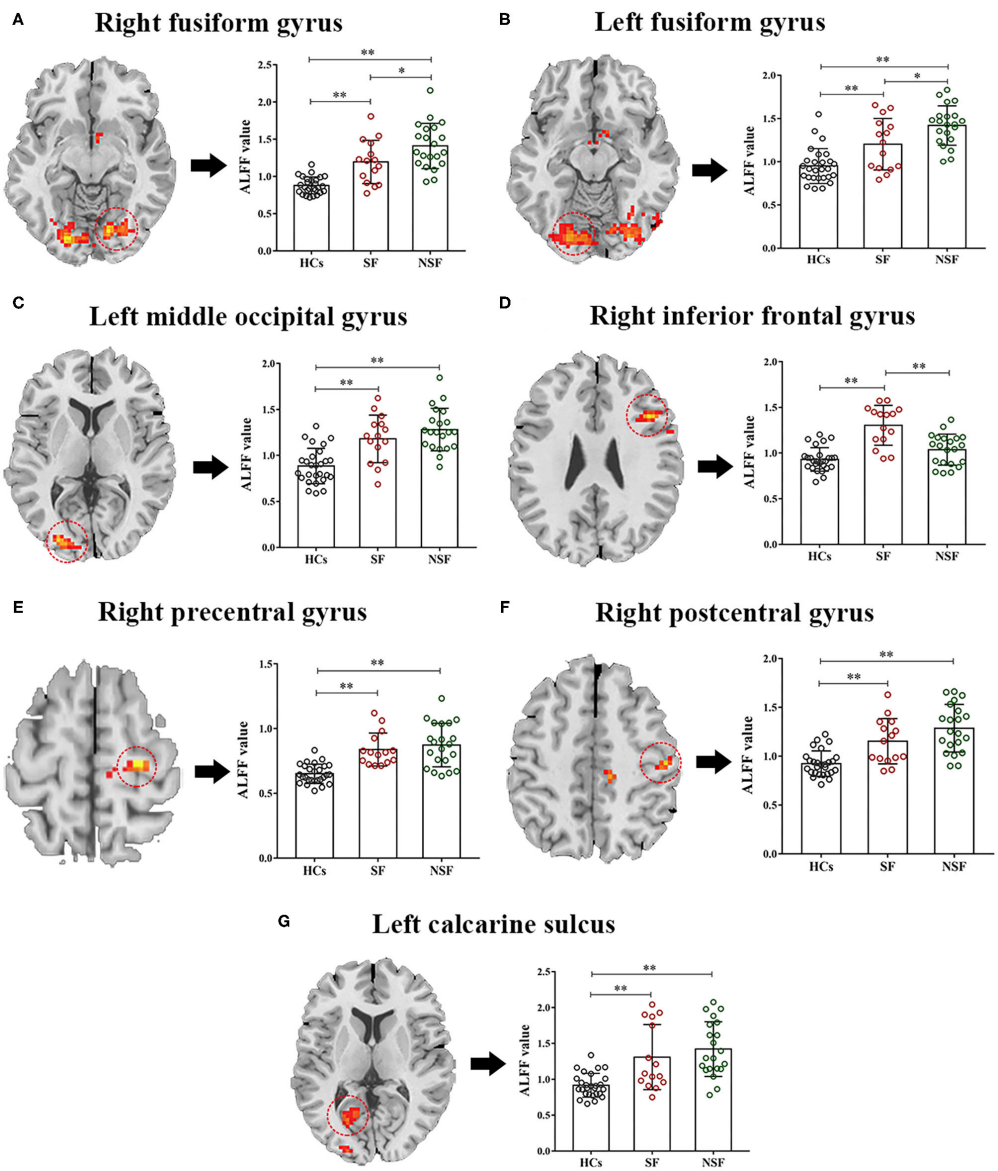
Pairwise comparison of ALFF in the seven brain regions. One-way ANOVA was used to compare the ALFF values of the three groups, and Bonferroni correction of *post-hoc* analysis was done at the same time in the significant brain regions **(A–G)**. **p < 0.01, *p < 0.05 after Bonferroni correction of *post-hoc* analysis. ALFF, the amplitude of low-frequency fluctuation; SF, seizure free; NSF, non-seizure free; HCs, healthy controls.

The values of ALFF were acquired by extracting signals at the significant clusters above and then were entered into pairwise comparison in SPSS. After Bonferroni correction of *post-hoc* analysis, all brain regions showed abnormal ALFF in the SF group, but only six brain regions showed abnormal ALFF in the NSF group relative to HCs. There were three abnormal brain regions for the SF and NSF. Specifically, the SF group showed increased ALFF in the bilateral FG, L.MOG, R.IFG, R.PreG, R.PostG, and L.CS (Table 2; Figure 2). The patients with NSF GTCS showed increased ALFF in the same region as the SF group, except for the R.IFG (Table 2; Figure 2). It was worth noting that a higher ALFF in the bilateral FG and lower ALFF in the R.IFG were found in the NSF group compared to the SF group (Figure 2). There were no significant differences in the L.MOG, R.PreG, R.PostG, and L.CS between the SF and NSF groups.

### Correlation Between ALFF and Clinical Characteristics

**Figure 3:**
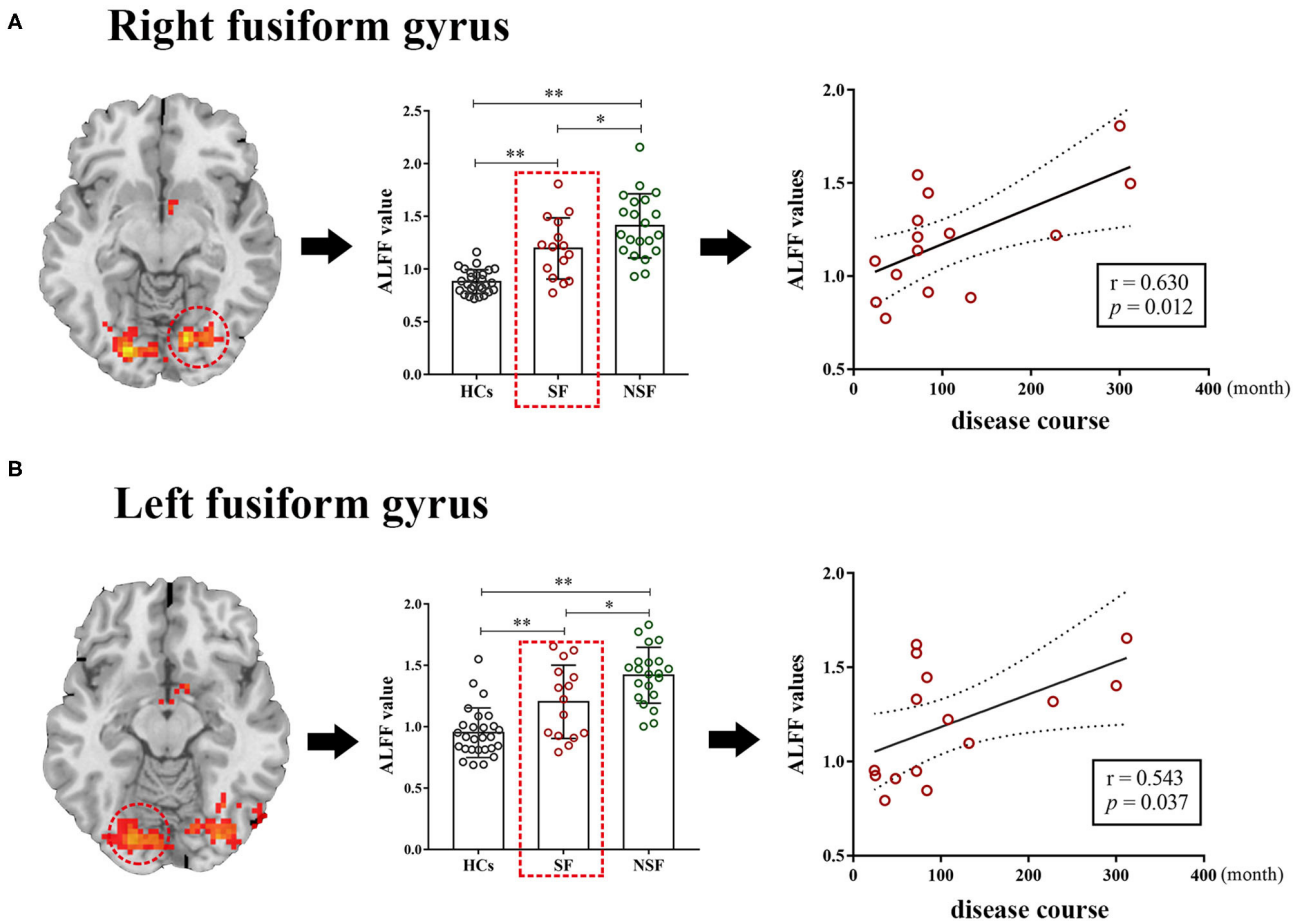
The correlation between the ALFF values and clinical characteristics. The ALFF values of the right fusiform gyrus (*r* = 0.630, *p* = 0.012) and left fusiform gyrus (*r* = 0.543, *p* = 0.037) were positively correlated with disease course in the SF group respectively **(A,B)**. ^**^p < 0.01, ^*^p < 0.05 after Bonferroni correction of post-hoc analysis. ALFF, the amplitude of low-frequency fluctuation; SF, seizure-free; NSF, non-seizure-free; HCs, healthy controls.

Correlation analyses between ALFF and clinical characteristics (disease course and age of onset of GTCS) were performed in the significant regions between the SF group (seven regions) and the NSF group (six region) separately. Among the total of 26 correlation analyses (13 abnormal regions, two clinical characteristics), two correlations reached *p* < 0.05, but none can survive Bonferroni correction. Specifically, two positive correlations were observed between ALFF in the R.FG (*r* = 0.630, *p* = 0.012) and L.FG (*r* = 0.543, *p* = 0.037) and disease course in the SF group, where patients with higher ALFF in these two brain regions had a longer course (Figure 3).

## Discussion

In the current study, we aimed to explore the underlying neural substrate of the different responses to adequate antiseizure drugs between the SF and NSF patients afflicted with GTCS through a cross-sectional study. We observed the changes of the spontaneous whole-brain activity through ALFF analysis among the SF, NSF, and HCs. A significant group effect was found in the R.FG, L.FG, L.MOG, R.IFG, R.PreG, R.PostG, and L.CS. The SF group showed increased ALFF in the bilateral FG, L.MOG, R.IFG, R.PreG, R.PostG, and L.CS, and the NSF group showed increased ALFF in the same regions as the SF group except the R.IFG. It was worth noting that higher ALFF in the bilateral FG was found in the NSF group compared to the SF group. Moreover, two positive correlations were observed between ALFF in the bilateral FG and disease course in the SF group.

Epilepsy is a complex neurological syndrome characterized by abnormally hypersynchronous hyperactivity of a population of neurons. Previous studies have suggested that the amplitude or power (square of amplitude) of low-frequency fluctuations can provide both the nature and extent of signal changes underlying spontaneous neuronal activities (31, 40). Generally, increased ALFF accompanied increased neuronal activity. In this study, we found that ALFFs of both the SF and NSF groups were significantly increased compared with those of HCs (except the R.IFG in the NSF group), indicating that neuronal excitability was significantly enhanced in patients with GTCS, which was consistent with the physiological mechanism of epilepsy. Abnormal functional changes in extensive brain regions were found in patients with GTCS according to previous studies (16, 41). For example, Ji and colleagues found that GTCS patients presented abnormal functional connectivity in many regions, such as the anterior cingulate cortex, inferior frontal gyrus, and bilateral cuneus (16). In this study, we found that patients with GTCS presented aberrant brain activity in the bilateral FG, L.MOG, R.IFG, R.PreG, R.PostG, and L.CS, which have also been reported in patients with IGE in previous studies (42–44).

It was worth noting that higher ALFF in the FG was found in the NSF group compared to the SF and HC groups. Previous EEG studies have shown that the slow-wave activity in epileptic regions was enhanced significantly, and the amplitude of slow wave was also increased prominently in intractable epilepsy patients (45, 46). As mentioned above, the characterization of spontaneous neuronal activities was based on the spontaneous fluctuations in the BOLD signals in widespread but functionally related brain regions (23). However, the correlation between slow-wave activity and ALFF was unclear, and there were some studies suggesting that ALFF may be a new biomarker for the physiological state of the brain (31). Therefore, over-enhanced ALFF in the FG seemed to be a novel neural substrate to interpret the occurrence of frequent seizures in NSF patients with GTCS in our study. The FG, located in the inferior temporal lobe, has been proposed to be a critical area for face processing and perception (47). Therefore, most of the previous studies focused on the relationship between the cognition deficit like visual naming or recognition performances and related brain regions like the FG in patients with epilepsy (48–50). However, some studies have shown that the FG was one of the epileptic foci and patients with epilepsy achieved a good seizure outcome with the resection of the FG (51, 52). In addition, abnormal structure and function were also found in the FG in patients with epilepsy (43, 53, 54). For example, increased sulcal depth in the FG in benign childhood epilepsy compared to the HCs (53) and decreased gray matter volume in the FG after temporal lobectomy in patients with mesial temporal lobe epilepsy who achieved seizure freedom (54) were found in recent studies. All this research suggested that the changes in the FG may be related to the clinical outcome of epilepsy. In our study, the NSF patients had higher ALFF in the FG compared with the SF group, which seems to once again verify the importance of the FG in the control of seizure in patients with GTCS.

On the other hand, two positive correlations were observed between ALFF in the bilateral FG and disease course in the SF group in the current study. Although the correlation analysis did not pass Bonferroni correction, it presented a certain correlation trend because the *p*-value reached 0.05. In other words, when the seizure is well controlled, the shorter course of the disease in patients with GTCS accompanied a lower ALFF in the bilateral FG, which may be more conducive to achieving seizure control. A previous study also indicated that epileptic patients with well-controlled seizure were more likely to be older, have late-onset epilepsy, and have shorter disease duration (55, 56). However, it was not clear why the course of the disease was associated with ALFF of the bilateral FG in the SF group but not in the NSF group. Our results seemed to show that the disease course was not related to the occurrence of seizures when patients failed to achieve seizure control. Indeed, in our study, we did not find statistical difference in the disease course between the SF and NSF groups. Therefore, beyond a certain range, the seizure control of patients with GTCS may be related to ALFF of the FG rather than the disease course. Of course, a larger sample size is needed to further verify the credibility due to insufficiently rigorous correlation results.

Some limitations in our study are worthy of consideration. First, the recruitment of participants was temporarily interrupted because of the sudden outbreak of COVID-19, and the relatively few participants may limit the statistical power. Future research with a larger sample size is needed to repeat the current experiment to ensure the credibility of the results. Second, patients with GTCS took different types of antiseizure drugs in our study, which may have different effects on brain activity as described in previous studies (57). Finally, this is a cross-sectional study which cannot provide longitudinal alteration data of the participants; thus, it cannot be determined whether abnormal ALFF in the FG could be used to predict clinical outcome in patients with GTCS. Follow-up studies are necessary to observe changes in ALFF before and after taking antiseizure drugs in patients with GTCS and their associations with the responses to antiseizure drugs. Given a positive result is found, the FG may become a new target for the treatment of NSF patients with GTCS. From this perspective, our study is of great significance in laying a foundation for further exploration.

## Conclusion

In summary, the study compared ALFF by rs-fMRI data among the SF group, NSF group, and HCs. Abnormal ALFF in the bilateral FG, L.MOG, R.IFG, R.PreG, R.PostG, and L.CS was found among three groups. Significantly higher ALFF in the bilateral FG was observed in the NSF group compared to the SF and HC groups. Our findings indicate that abnormal brain activity in the FG may be one potential neural substrate to interpret the failure of seizure control in patients with GTCS.

## Data Availability Statement

The original contributions presented in the study are included in the article/Supplementary Materials, further inquiries can be directed to the corresponding author/s.

## Ethics Statement

The studies involving human participants were reviewed and approved by the Anhui Medical University Ethics Committee. The patients/participants provided their written informed consent to participate in this study.

## Author Contributions

MZ and LF made substantial contributions to design study. MH, XC, and LW were responsible for subject recruitment, case diagnosis, and fMRI data acquisition and analysis. MZ, JZ, and ZD contributed to statistical analysis and interpretation of data. BQ provided the machine for us to collect fMRI data. MZ, LF, and YW were responsible for drafting the manuscript and revising it. All authors contributed to the article and approved the submitted version.

## Funding

This work was supported by Natural Science grants to YW (Grant Number: 81671290) from the National Natural Science Foundation of China.

## Conflict of Interest

The authors declare that the research was conducted in the absence of any commercial or financial relationships that could be construed as a potential conflict of interest. The reviewer JZ declared a shared affiliation, with no collaboration, with the authors MZ, LF, MH, XC, JZ, ZD, and YW at the time of the review.

## Publisher's Note

All claims expressed in this article are solely those of the authors and do not necessarily represent those of their affiliated organizations, or those of the publisher, the editors and the reviewers. Any product that may be evaluated in this article, or claim that may be made by its manufacturer, is not guaranteed or endorsed by the publisher.
